# Huntingtin in the amygdaloid basolateral complex is correlated with Vonsattel staging in Huntington’s disease

**DOI:** 10.1093/braincomms/fcaf502

**Published:** 2025-12-22

**Authors:** Pablo Sanchez-Migallon, Alicia Flores-Cuadrado, Patricia Villanueva-Anguita, Alberto Rabano, Julia Vaamonde, Daniel Saiz-Sanchez, Alicia Mohedano-Moriano, Veronica Astillero-Lopez, Carmen Soriano-Herrador, Alino Martinez-Marcos, Isabel Ubeda-Banon

**Affiliations:** Neuroplasticity and Neurodegeneration Group, IB-UCLM, Ciudad Real Medical School, University of Castilla-La Mancha, 13071 Ciudad Real, Spain; Neuroplasticity and Neurodegeneration Group, Instituto de Investigación Sanitaria de Castilla-La Mancha (IDISCAM), 13071 Ciudad Real, Spain; Neuroplasticity and Neurodegeneration Group, IB-UCLM, Ciudad Real Medical School, University of Castilla-La Mancha, 13071 Ciudad Real, Spain; Neuroplasticity and Neurodegeneration Group, Instituto de Investigación Sanitaria de Castilla-La Mancha (IDISCAM), 13071 Ciudad Real, Spain; Neuroplasticity and Neurodegeneration Group, IB-UCLM, Ciudad Real Medical School, University of Castilla-La Mancha, 13071 Ciudad Real, Spain; Neuropathology Department, Alzheimer’s Disease Research Unit, CIEN Foundation, CIBERNED, Institute of Health Carlos III, Queen Sofía Foundation Alzheimer Research Center, 28031 Madrid, Spain; Neuroplasticity and Neurodegeneration Group, IB-UCLM, Ciudad Real Medical School, University of Castilla-La Mancha, 13071 Ciudad Real, Spain; Neurology Service, Ciudad Real University Hospital, 13005 Ciudad Real, Spain; Neuroplasticity and Neurodegeneration Group, IB-UCLM, Ciudad Real Medical School, University of Castilla-La Mancha, 13071 Ciudad Real, Spain; Neuroplasticity and Neurodegeneration Group, Instituto de Investigación Sanitaria de Castilla-La Mancha (IDISCAM), 13071 Ciudad Real, Spain; Neuroplasticity and Neurodegeneration Group, Instituto de Investigación Sanitaria de Castilla-La Mancha (IDISCAM), 13071 Ciudad Real, Spain; Faculty of Health Sciences, University of Castilla-La Mancha, 45600 Talavera de la Reina, Spain; Neuroplasticity and Neurodegeneration Group, IB-UCLM, Ciudad Real Medical School, University of Castilla-La Mancha, 13071 Ciudad Real, Spain; Neuroplasticity and Neurodegeneration Group, Instituto de Investigación Sanitaria de Castilla-La Mancha (IDISCAM), 13071 Ciudad Real, Spain; Neuroplasticity and Neurodegeneration Group, IB-UCLM, Ciudad Real Medical School, University of Castilla-La Mancha, 13071 Ciudad Real, Spain; Neuroplasticity and Neurodegeneration Group, Instituto de Investigación Sanitaria de Castilla-La Mancha (IDISCAM), 13071 Ciudad Real, Spain; Neuroplasticity and Neurodegeneration Group, IB-UCLM, Ciudad Real Medical School, University of Castilla-La Mancha, 13071 Ciudad Real, Spain; Neuroplasticity and Neurodegeneration Group, Instituto de Investigación Sanitaria de Castilla-La Mancha (IDISCAM), 13071 Ciudad Real, Spain; Neuroplasticity and Neurodegeneration Group, IB-UCLM, Ciudad Real Medical School, University of Castilla-La Mancha, 13071 Ciudad Real, Spain; Neuroplasticity and Neurodegeneration Group, Instituto de Investigación Sanitaria de Castilla-La Mancha (IDISCAM), 13071 Ciudad Real, Spain

**Keywords:** amygdala, glia, human, sexual dimorphism, stereology

## Abstract

Huntington’s disease has traditionally been considered a motor disorder, but it is currently classified as a multisystem neurodegenerative disease that involves brain regions, such as the amygdala, and causes depression. The aim of the present study was to analyse the distribution of huntingtin in the human amygdaloid basolateral complex, considering its nuclei, sex, triplet repeats and Vonsattel score, as well as to characterize the cellular relationships between huntingtin and associated copathologies. The present study included 23 human brain samples from patients (males and females) with and without Huntington’s disease, Parkinson’s disease and Alzheimer’s disease. An unbiased stereology approach was used to quantify huntingtin deposits. Multiple immunofluorescence experiments were conducted to analyse the relationship between huntingtin and glial populations. Immunohistochemistry against pathological markers of other neurodegenerative diseases was also carried out. Quantification data did not reveal differences among different nuclei (basomedial, basolateral or lateral) in the basolateral complex or according to sex. Huntingtin deposits did not correlate with cytosine–adenine–guanine (CAG) repeats. However, these deposits were positively correlated with pathological Vonsattel grades. Additional aggregates of other pathological proteinopathies were also observed. This correlation between the human basolateral amygdaloid complex and the Vonsattel stage provides a new perspective for neuropathological diagnosis and helps in understanding nonmotor symptoms such as depression.

## Teaser

Sanchez-Migallon *et al*. report that huntingtin deposits in the human amygdaloid basolateral complex positively correlate with Vonsattel neuropathological grades. This provides a new perspective for neuropathological diagnosis and helps in understanding nonmotor symptoms such as depression.

## Graphical Abstract

**Graphical Abstract:**
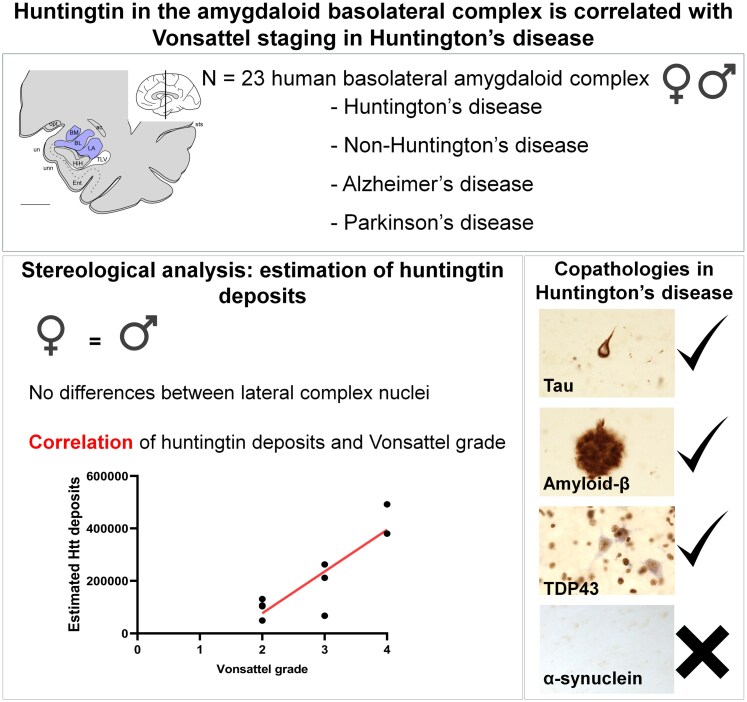

## Introduction

Huntington’s disease is a rare disease^1^ that involves motor, behavioural and cognitive manifestations caused by an autosomal dominant expansion of the cytosine–adenine–guanine (CAG) trinucleotide repeat in the huntingtin (HTT) gene on chromosome 4. This mutation mostly causes neurodegeneration in the striatal medium spiny neurones of the caudate nucleus and putamen.^2^ Diagnosis is based on motor signs and genetic background,^3^ which is crucial for obtaining a complete view.^4^ For neurodegenerative diseases,^5-7^ particularly for Huntington’s disease, current therapies mostly treat symptoms,^8^ but stem cell–based cell therapy appears to be promising,^9^ with antisense oligonucleotides (ASOs),^10^ peptide inhibitors and clustered regularly interspaced short palidromic repeats and enzyme Cas9 (CRISPR/Cas9) gene editing likely being the most promising.^11^

The traditional dogma of Huntington’s disease pathogenesis is based on the gain of function (toxicity) of the mutant HTT protein, which kills neurones; however, the loss of function of wild-type HTT may also influence pathology since it is a vital gene for brain development.^12^ Aberrant mutant protein activity includes excitotoxicity, mitochondrial dysfunction, disruption of proteostasis, dopamine imbalance, apoptotic pathways, transcriptional dysregulation and neuroinflammation via the activation of astrocytes and microglia.^13^ Whether mutant HTT inclusions are toxic or neuroprotective is uncertain,^13^ but accumulating evidence indicates that this proteinopathy may act in a prion-like manner.^14^

Striatal involvement with macroscopic and microscopic neuronal loss and gliosis allowed the establishment of neuropathological 0–4 staging.^15^ This classical staging, however, is based on macroscopic (striatal volume loss) and microscopic (neurodegeneration and gliosis assessed by methods such as haematoxylin and eosin and cresyl violet) changes that currently appear somewhat limited. In fact, neuroimaging studies have revealed that beyond striatal involvement, cortical and subsequently cortico-striatal connections are particularly susceptible to disease pathology.^16,17^ In the same vein, mutant HTT deposits, identified via anti-ubiquitin, anti-p62^18-20^ and anti-HTT^21^ antibodies, demonstrate pathology beyond the striatum. Involvement was not limited to the caudate and putamen but also affected the cerebral cortex, and cortical HTT inclusion density correlated with CAG repeat expansion but not with Vonsattel staging.^21^ Accumulating evidence suggests that HTT is involved in the skin,^22^ liver^23^ and gut.^24^

Currently, Huntington’s disease is considered a multisystem neurodegenerative disease with crucial brain regions involved in symptomatology, such as the amygdala,^25-27^ which has been neuropathologically observed only sporadically,^28^ and a systematic analysis is needed.^20^ Functionally^29^ and structurally, the human amygdala is among the most intricate, heterogeneous and complex brain structures closely connected to the high-order association cortex, which is highly relevant in disorders such as anxiety and depression.^30,31^

HTT deposits are not routinely used for the analysis of pathological progression, and critical regions such as the amygdala have often been overlooked. Therefore, the aim of the present study was to perform the first characterization of HTT pathology in the amygdaloid basolateral complex.

An unbiased stereology approach was used to compare the nuclei of the complex and the dependence on the sex of the patient. The correlations between the number of CAG triplet repeats and the Vonsattel stage were analysed. The cellular relationships of HTT, including glial populations, have also been studied. Finally, the occurrence of other proteinopathies has also been investigated.

## Materials and methods

### Ethics approval and patient consent statement

Postmortem human brain samples were collected and processed following standard operating procedures and the appropriate approval of ethical and scientific committees. All experiments were authorized by the Clinical Research Ethics Committee of Ciudad Real University Hospital (SBPLY/21/180501/000093). These processes included obtaining the donors’ written consent. All of this ensures compliance with the Helsinki Declaration.

### Human samples

**Figure 1:**
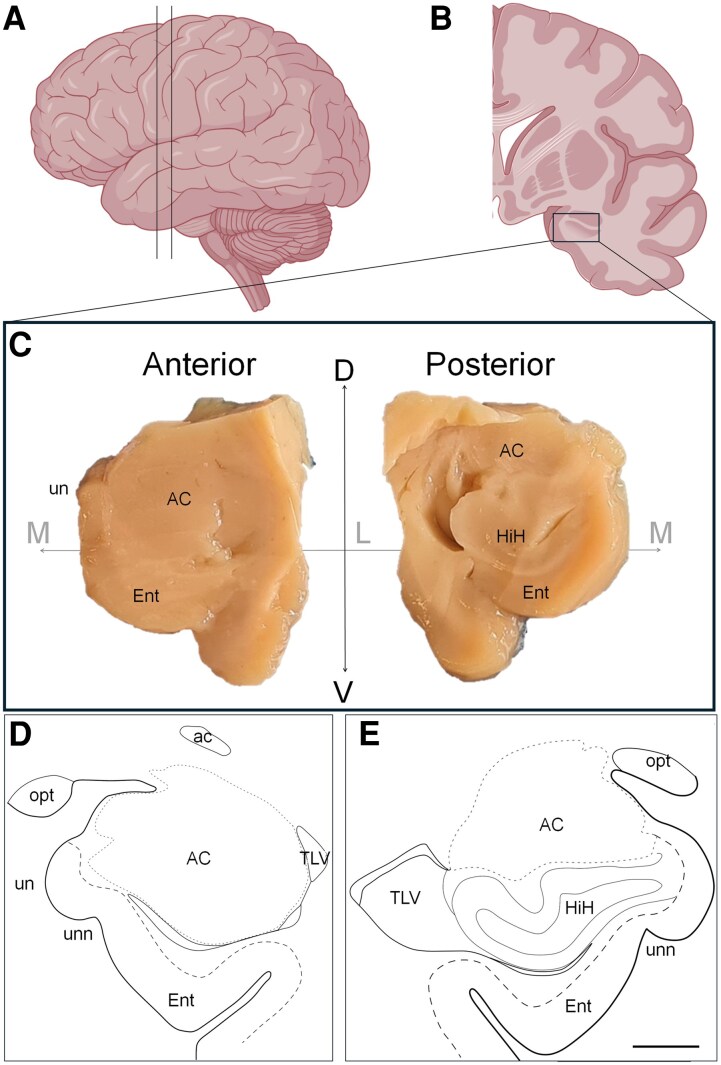
**Handling and orientation of the samples.** Following standardized protocols in the tissue bank, the left hemisphere of the brain was fixed and processed for neuropathological study. Coronal slices of a maximum thickness of 1 cm were made from the frontal pole to the occipital pole (**A**), and the regions of interest requested by the researcher were carved (**B**). Amygdala samples of ∼2 × 2 cm were obtained in the laboratory and were photographed and anatomically oriented (**C**) with the help of a human brain atlas (**D–E**). Scale bar (**C–E**) = 0.5 cm. ac, anterior commissure; AC, amygdaloid complex; D, dorsal; Ent, entorhinal cortex; HiH, hippocampus; L, lateral; M, medial; opt, optic tract; TLV, temporal horn of lateral ventricle; un, uncus; unn, uncal notch; V, ventral. **A** and **B** were created in BioRender. Saiz-sánchez, D. (2025) https://BioRender.com/7hk7e5c (agreement number: JA291Q1CY3).

The samples and data from the donors included in this study were provided by the BTCIEN, which is integrated into the Spanish National Biobank Network. Following standard operating procedures, the tissue was divided into two halves via a mid-sagittal cut after brain extraction. The right half was frozen immediately and stored long term at −80°C, whereas the left half was fixed in formaldehyde for subsequent neuropathological diagnosis.^32^ Blocks of left formalin-fixed amygdalae that were oriented and anatomically identified were obtained from this brain bank^33^ (Fig. 1). The present study included 13 human brain samples from patients with and without Huntington’s disease (Table 1). For cryoprotection, the tissue was immersed for 48 h at 4°C in a phosphate-buffered (PB) solution of 2% dimethyl sulfoxide (DMSO) and 10% glycerol and for 48 h at 4°C in a PB solution of 2% DMSO and 20% glycerol. Coronal sections (50 μm) were obtained via a freezing microtome and were collected in an orderly manner in 13 series (section evaluation interval in Supplementary Tables 1 and 2). The first series was used for Nissl staining, and the remaining series were stored in cryoprotective solution at −20°C until further processing.

**Table 1:** Demographic and clinicopathological features of the individuals used in the present study

Case	Sex	Age (years)	Brain weight (grams)	Vonsattel grade	Number of CAG repeats	Clinical diagnosis	Neuropathological diagnosis
1	M	72	1275	2	19/44^a^	HD	HD. Braak tau II. Score vascular 2
2	M	72	1124	3	20/43^b^	HD	HD. Braak tau III. Score vascular 3
3	M	70	n.a.	3	n.a.	HD	HD
4	F	72	n.a.	2	n.a.	HD	HD. Braak II
5*	M	70	1111	2	n.a.	HD	HD. Braak tau II. Score vascular 4
6	F	72	n.a.	2	19/44^b^	HD	HD. Braak tau I
7	M	64	1025	4	19/44^b^	HD	HD. Braak tau I. Score vascular 6
8	F	68	945	3	28/43^a^	HD	HD. Braak tau I. Score vascular 5
9	M	53	939	2	/45^a^	HD	HD. Score vascular 2
10**	F	56	950	4	18/45^a^	HD	HD
11	M	62	960	4	22/41^a^	HD	HD
12**	M	57	1228	2	/39^a^	HD	HD. Braak tau II
13	M	88	1285	-	-	Control	Control. Braak tau II
14	M	59	1400	-	-	Control	Control
15	M	56	1400	-	-	Control	Control. Braak tau I
16	M	43	1412	.	.	Control	Control. Braak tau 0
17	M	58	944	-	-	Control	Control. Braak tau 0
18	H	58	1500	-	-	Control	Control. Braak tau I
19	F	65	1050	-	-	Control	Control. Braak tau II
20	F	81	1100	-	-	Control	Control. Braak tau II
21	F	82	1300	-	-	PD	PD. Braak syn 5
22	F	71	1006	-	-	AD	AD. Braak tau V; Braak syn 0
23	F	89	910	-	-	AD	AD. TDP43 + in amygdala. Braak tau V

AD, Alzheimer’s disease; F, female; HD, Huntington’s disease; M, male; n.a., not available; PD, Parkinson’s disease.

^a^Genetic testing on premortem blood.

^b^Genetic testing on postmortem brain tissue. *Outliers, **used for the development of the methodology.

### Immunohistochemistry and immunofluorescence

The tissue was boiled under pressure for 2 min and 30 s in citrate buffer to determine its antigenicity. Endogenous peroxidase activity was inhibited by incubation in a 30-min bath in 1% H_2_O_2_ in PBS. The sections were incubated with primary antibodies and subsequently incubated for 2 h with secondary antibodies (Supplementary Table 3). For immunohistochemistry, the sections were incubated in an avidin–biotin complex (ABC standard, Vector, containing 0.3% TX-100) and reacted with 0.025% 3,3′-diaminobenzidine and 0.1% H_2_O_2_. The sections were mounted, dried and coverslipped with dioxane, phenol, xylene (DPX) (Sigma–Aldrich). For immunofluorescence, the sections were mounted, dried and coverslipped with polyvinyl alcohol mounting medium (PVA-DABCO) (Sigma–Aldrich).

### Stereological quantification

**Figure 2:**
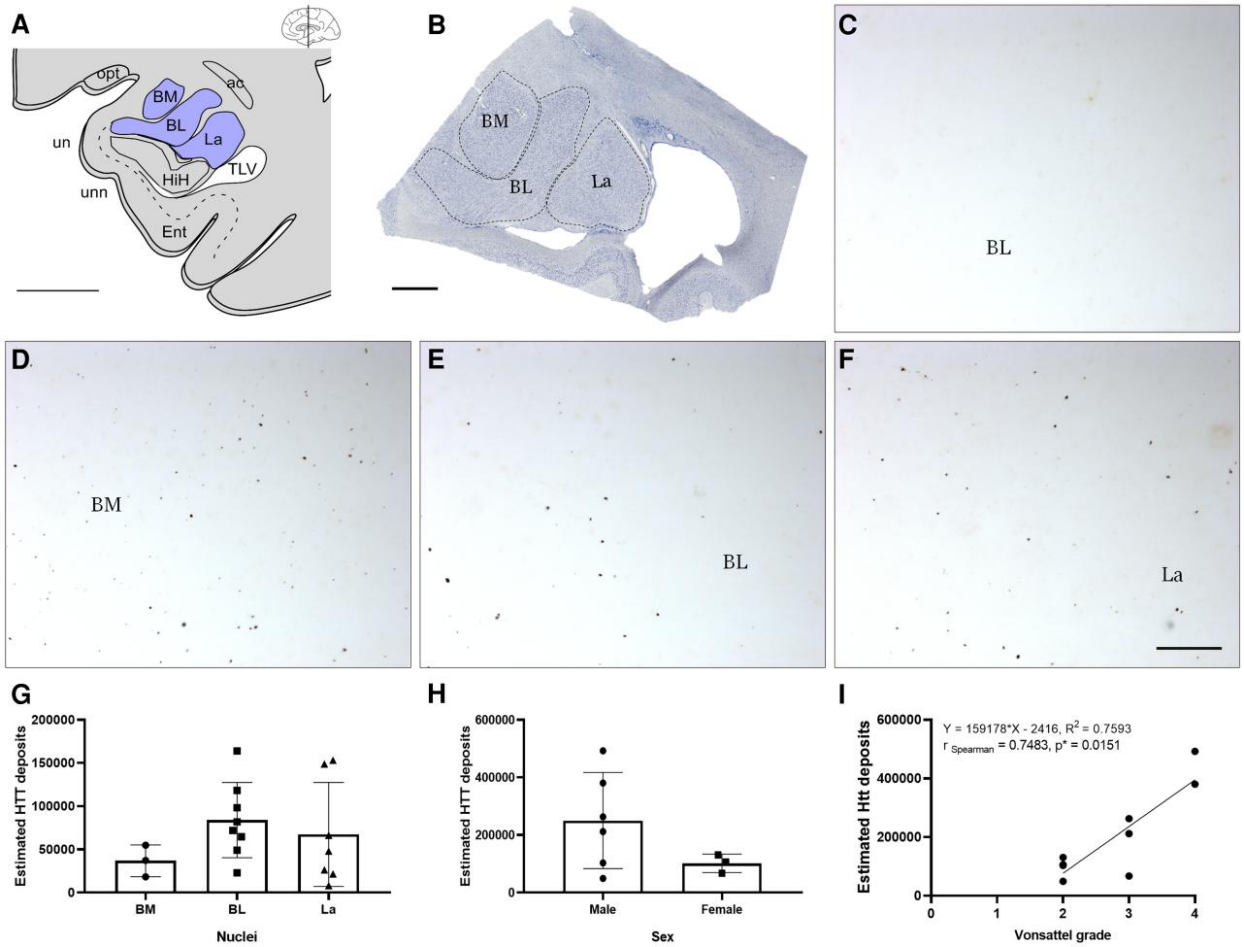
**HTT distribution in the human amygdaloid basolateral complex.** (**A**) Human amygdala location diagram; scale bar = 1000 μm. (**B**) Mosaic reconstruction of Nissl-stained coronal sections illustrating the human amygdaloid basolateral complex; scale bar = 1000 μm. Images illustrating immunostaining for HTT in the basolateral nucleus in control (**C**) and pathological patient samples (**D–F**); scale bar (**C–F**) = 20 μm. (**G and H**) Graphs illustrating the means ± SDs of the estimated numbers of HTT deposits; statistical comparisons were performed via one-way ANOVA (Tukey *post hoc* test) and two-tailed *t*-tests (*N* = 18 and *N* = 9, respectively), and (**I**) correlations with Vonsattel stages were performed; Spearman’s test was used for correlation analyses (*N* = 9). Dots in **G–I** represent data-point of each case analysed. ac, anterior commissure; BL, basolateral amygdaloid nucleus; BM, basomedial amygdaloid nucleus; Ent, entorhinal cortex; HiH, hippocampus; La, lateral amygdaloid nucleus; opt, optic tract; TLV, temporal horn of lateral ventricle; un, uncus; unn, uncal notch.

Stereological analyses were performed via Stereo Investigator software (MBF Bioscience, 185 Allen Brook Lane, Suite 101 Williston, VT 05495, USA) coupled with a Zeiss Axio Imager M2 microscope (Carl-Zeiss-Straße 22, 73447 Oberkochen, Germany). The cases included in the analyses met the statistical (outliers) and stereological (number of sections) criteria (Supplementary Tables 1 and 2). In the stereological methods, the optical fractionator probe enables the sampling of a 3D region of interest and estimation of the total number of particles.^34^ The estimated amount of HTT deposits was calculated via this probe (Plan Apochromat, 63×/1.4, oil lens, Ref. 420782-9900). In the human amygdala (Fig. 2A), under low magnification, the basomedial (BM), basolateral (BL) and lateral (La) nuclei of the basolateral complex^35^ were identified and outlined (Plan-Neofluar 1×/0.025; Ref. 420300-9900) (Fig. 2B).^33,36^ The parameters used were X = 75 × Y = 75 µm counting frame size, 2 µm guard zone, 5 µm height dissector and X = 200 × Y = 200 µm sampling grid size (Supplementary Tables 1 and 2).

### Statistical analysis

Statistical analysis was performed with GraphPad Prism® software (v8.0.1; La Jolla, CA). Normality and outliers were analysed via the Shapiro–Wilk test (*N* < 50) and the ROUT method (Q = 1%), respectively. Statistical comparisons were performed via two-tailed *t*-tests and one-way ANOVA (Tukey *post hoc* test). The data are presented as the means ± standard deviations (SDs). Spearman’s test was used for correlation analyses. Differences were regarded as statistically significant at *P* < 0.05.

## Results

HTT deposits were quantified in the BL complex in both sexes and correlated with the number of CAG repeats and Vonsattel grade. Its relationships with glial cells were analysed, as well as its concurrence with other pathologies.

### Huntingtin distribution in the human amygdaloid basolateral complex

The distribution of HTT deposits was stereologically quantified among the different components of the human amygdaloid basolateral complex. As expected, labelling was absent in nondiseased cases (Fig. 2C) and observable in tissues from Huntington’s disease patients in the BM (Fig. 2D), BL (Fig. 2E) and La (Fig. 2F). The data, including the number of sections, counting frame area, sampling grid area, number of sampling sites, total number of markers counted, estimated population and coefficient of error (Gundersen), are available in Supplementary Tables 1 and 2.

One-way ANOVA did not reveal differences in the estimated number of HTT deposits in different nuclei (F(2, 15) = 1.194, *P* = 0.3303) (Fig. 2G). One-way ANOVA did not reveal a difference in the density (HTT deposits/mm^3^) of HTT deposits between different nuclei (F(2, 15) = 0.7012, *P* = 0.5115) (results not shown).

### Huntingtin distribution in the human amygdaloid basolateral complex by sex

Sexual dimorphism was also analysed. The male and female groups included in the study (Table 1) were not significantly different in terms of age (unpaired two-tailed *t*-test, t7 = 1.143; *P* = 0.2907) or Vonsattel stage (unpaired two-tailed *t*-test, t7 = 1.155; *P* = 0.2861). Analysis of the distribution of HTT among sexes also revealed no significant differences (unpaired two-tailed *t*-test, t7 = 1.477; *P* = 0.1832) (Fig. 2H). No difference was observed in terms of density (unpaired two-tailed *t*-test, t7 = 0.7192; *P* = 0.4953) (results not shown).

### Correlations of huntingtin deposits, cytosine–adenine–guanine repeats and pathological stage

The estimated HTT deposits do not correlate with the number of CAG repeats (Spearman *r* = −0.4304; *P* (one-tailed) = 0.1738) (not shown). No correlation was detected between density and the number of CAG repeats (Spearman *r* = −0.2620; *P* (one-tailed) = 0.2833) (results not shown). With respect to the sex, it is not possible to analyse females because of the low case number. In males, there was no correlation (Spearman *r* = − 0.6669; *P* (one-tailed) = 0.1333) (results not shown).

However, a significant positive correlation was found between total HTT deposits in the human amygdaloid BL complex and the Vonsattel grade (Spearman *r* = 0.7483; *P* (one-tailed) = 0.0151) (Fig. 2I). By sex, this correlation is not maintained in females—keeping in mind that the number of cases is quite low (Spearman *r* = −0.8660; *P* (one-tailed) = 0.333), but it is maintained in males (Spearman *r* = 0.9562; *P* (one-tailed) = 0.0111; *r*^2^ = 0.9318; Y = 180134 * X − 290439). With respect to density, there was no correlation between density and the Vonsattel grade (Spearman *r* = 0.3742; *P* (one-tailed) = 0.1595) in either females (Spearman *r* = −0.8660; *P* (one-tailed) = 0.333) or males (Spearman *r* = 0.7171; *P* (one-tailed) = 0.0889) (results not shown).

### Relationship between huntingtin deposits and cell type

**Figure 3:**
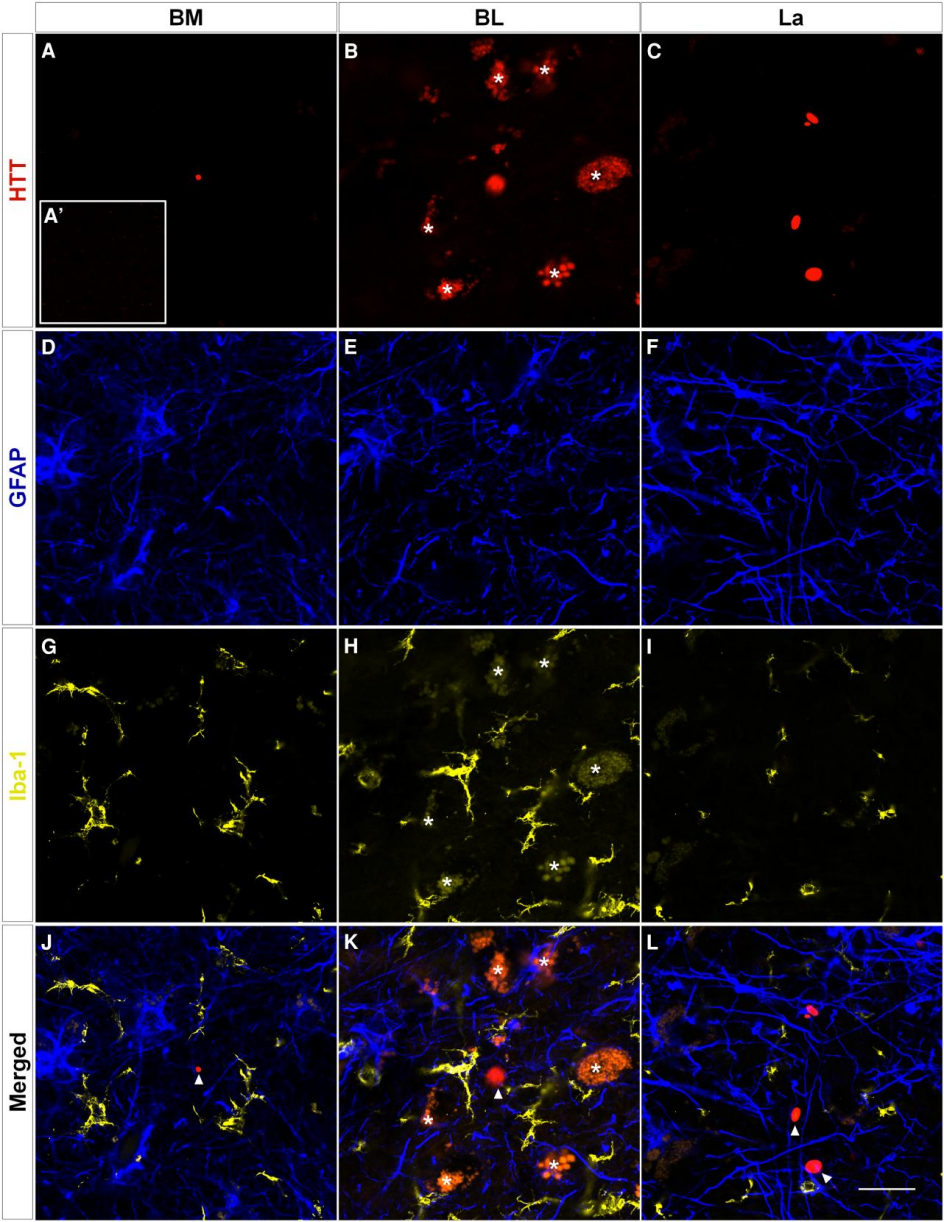
**HTT distribution and gliosis.** Triple immunofluorescence in the human basolateral amygdaloid complex in Huntington’s disease: BM (**A, D, G and J**), BL (**B, E, H and K**), and La (**C, F, I and L**) nuclei. An image of the control tissue is included (**A′**). The relationships between HTT deposits (**A–C**; arrowheads in **J–L**) and astroglia (GFAP) (**D–J, K and L**) and microglia (Iba-1) (**G–L**) are shown. Asterisks (**B, H and K**) indicate lipofuscin labelling. Scale bars (**A–L**) = 20 μm and (**A′**) = 150 μm.

Since gliosis is among the most relevant microscopic traits in the neuropathological diagnosis of Huntington’s disease, multiple immunofluorescence experiments were performed, in which specific antibodies against HTT, glial fibrillary acidic protein (GFAP) (astroglia) and Iba1 (microglia) (Supplementary Table 3) were combined for qualitative description. Confocal microscopy revealed, among the different BL complex nuclei, HTT deposits (Fig. 3A–C) and different patterns of astrogliosis (Fig. 3D–F) and microgliosis (Fig. 3G–I), but no colocalization of HTT deposits with either microglial or astroglial populations was detected (arrowheads in Fig. 3J–L). Asterisks (Fig. 3B, H and K) indicate lipofuscin labelling. HTT labelling was absent in the controls (Fig. 3A′).

### Copathologies

**Figure 4:**
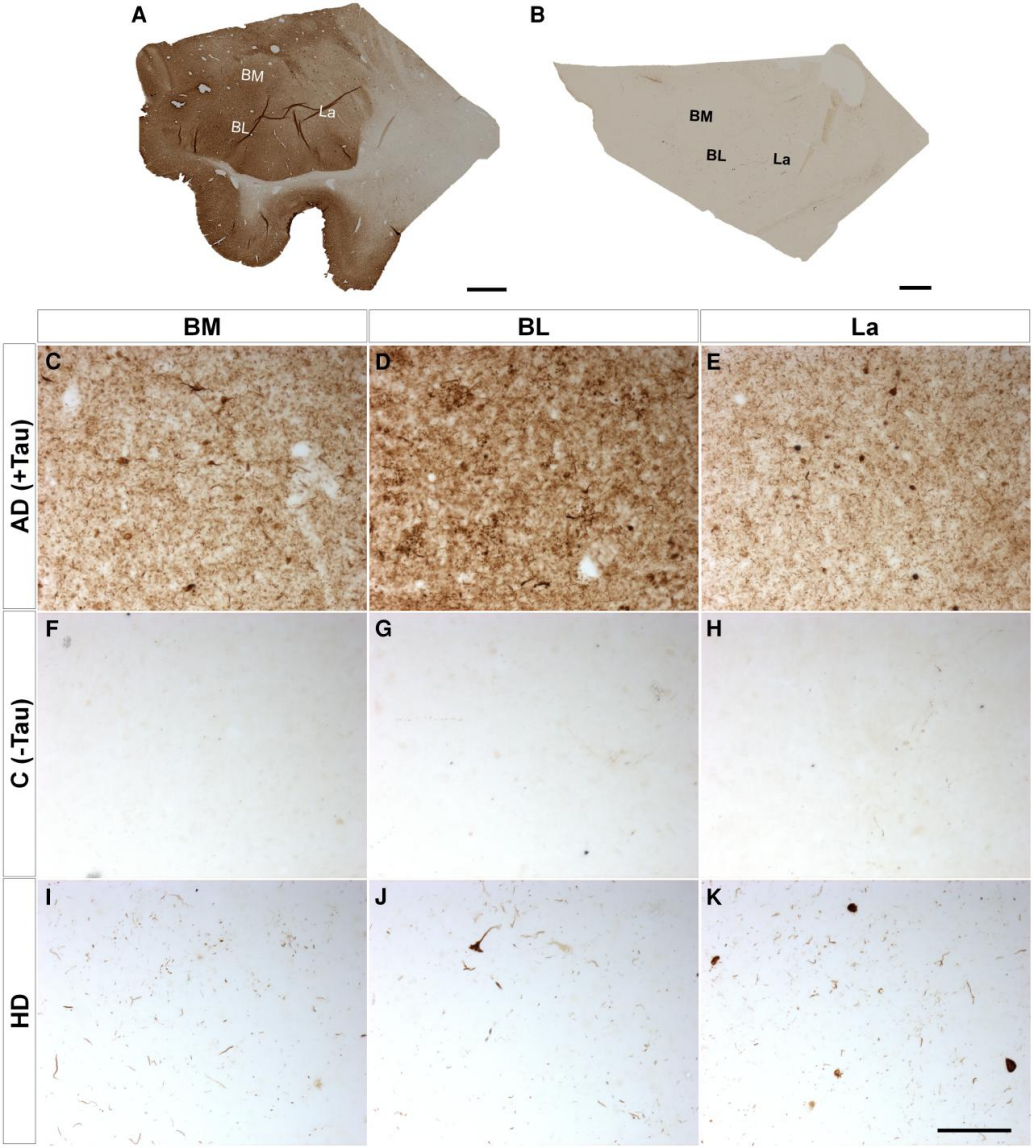
**Tau copathology.** Mosaic reconstruction of tau immunohistochemically reacted coronal sections of the human amygdala BL complex from samples from patients with Alzheimer’s disease (**A**) and Huntington’s disease (**B**); scale bar = 2000 μm. Coronal sections of human BM (**C, F and I**), BL (**D, G and J**) and La (**E, H and K**) amygdaloid nuclei immunohistochemically stained for tau protein from samples from patients with Alzheimer’s disease (control positive) (**C–E**), without disease (negative control) (**F–H**) and with Huntington’s disease (**I–K**). Scale bar (**C–K**) = 100 µm.

**Figure 5:**
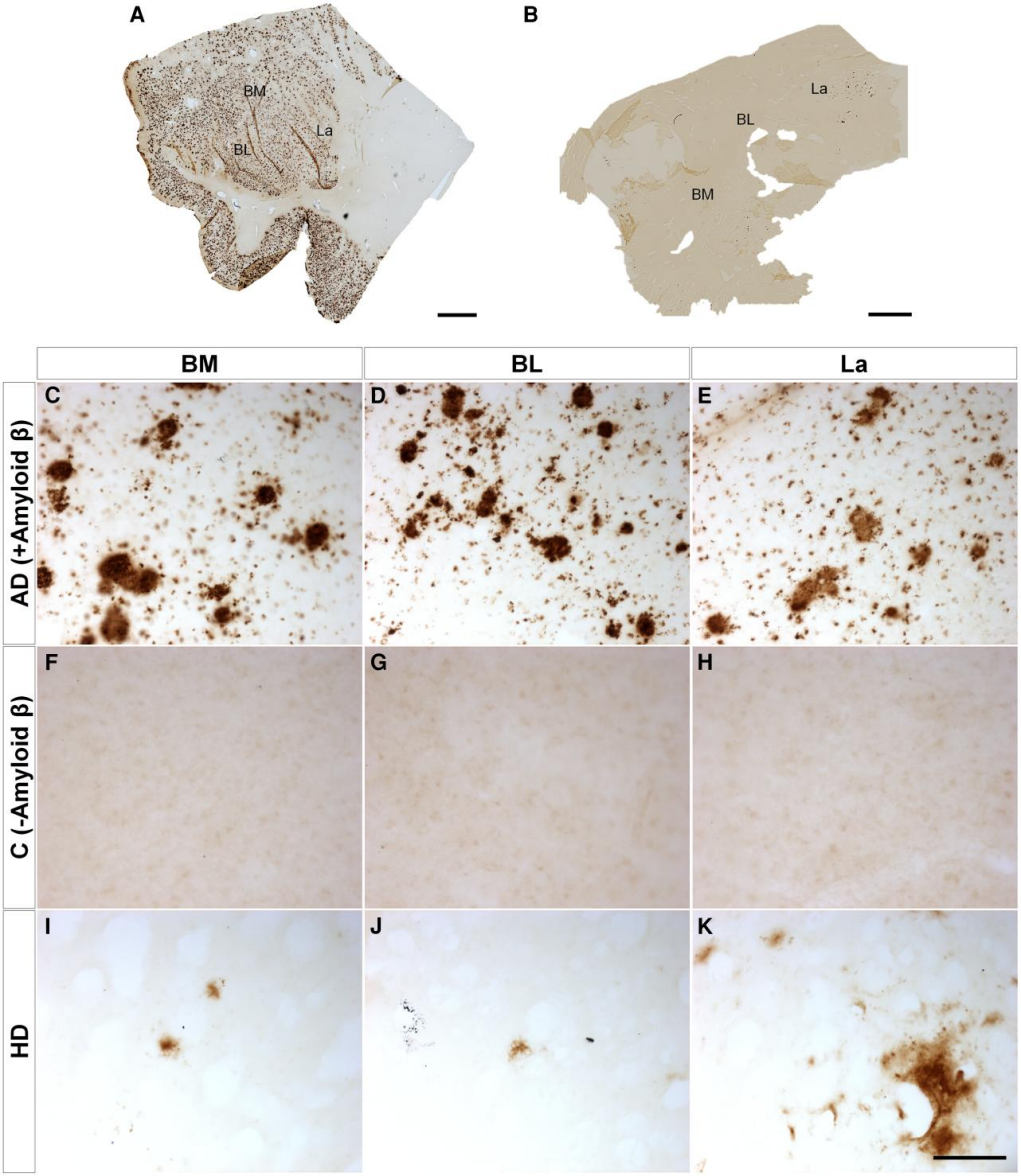
**Amyloid β copathology.** Mosaic reconstruction of amyloid β immunohistochemically reacted coronal sections of the human amygdala BL complex from samples from patients with Alzheimer’s disease (**A**) and Huntington’s disease (**B**); scale bar = 2000 μm. Coronal sections of human BM (**C, F and I**), BL (**D, G and J**) and La (**E, H and K**) amygdaloid nuclei immunohistochemically stained for amyloid β protein from samples from patients with Alzheimer’s disease (control positive) (**C–E**), without disease (negative control) (**F–H**) and with Huntington’s disease (**I–K**). Scale bar (**C–K**) = 100 µm.

**Figure 6:**
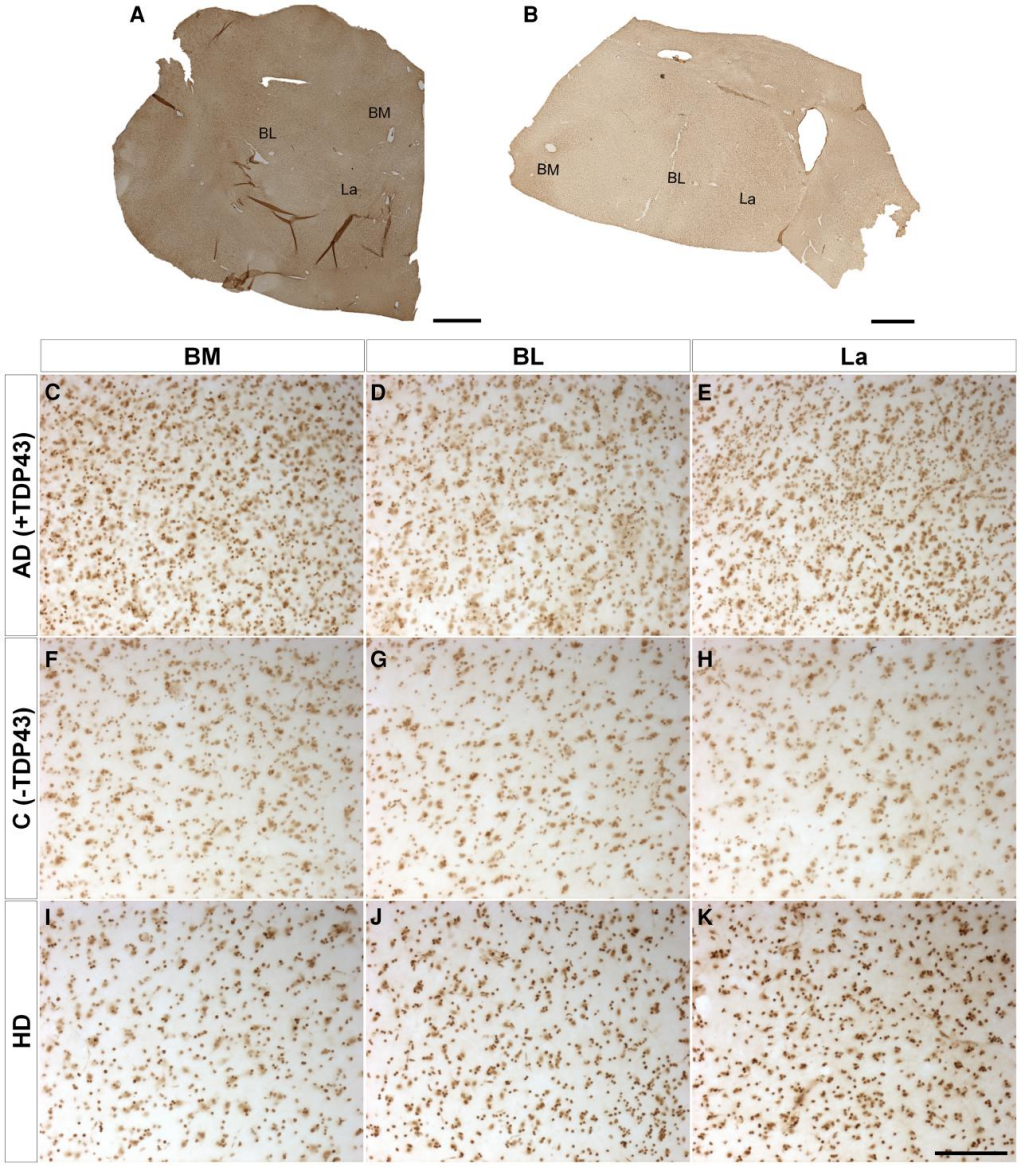
**TDP43 copathology.** Mosaic reconstruction of TDP43 immunohistochemically reacted coronal sections of the human amygdala BL complex from samples from patients with Alzheimer’s disease (**A**) and Huntington’s disease (**B**); scale bar = 2000 μm. Coronal sections of human BM (**C, F and I**), BL (**D, G and J**) and La (**E, H and K**) amygdaloid nuclei immunohistochemically stained for TDP43 protein from samples from patients with Alzheimer’s disease (control positive) (**C–E**), without disease (negative control) (**F–H**) and with Huntington’s disease (**I–K**). Scale bar (**C–K**) = 100 µm.

**Figure 7:**
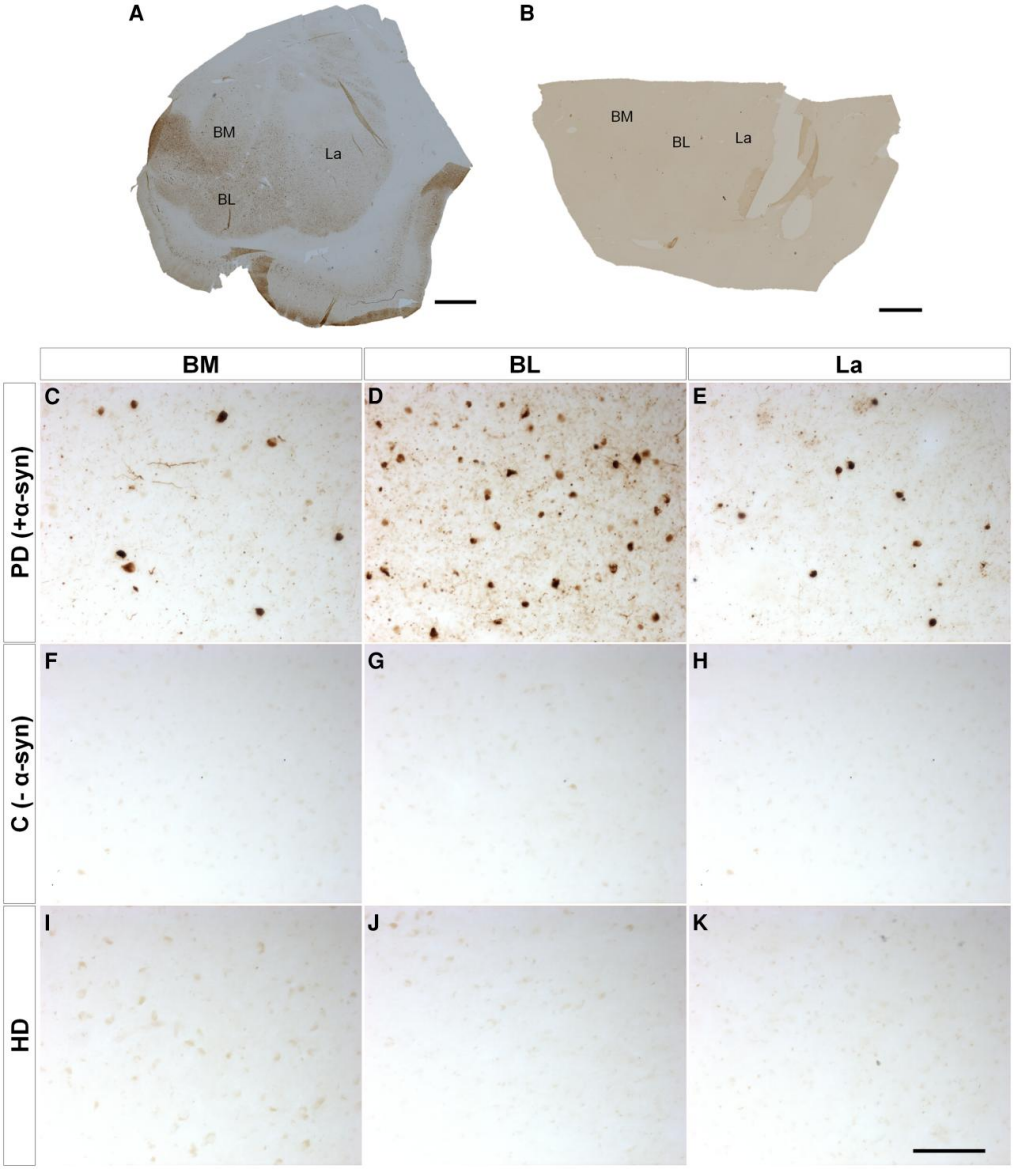
**α-Synuclein (α-syn) copathology.** Mosaic reconstruction of α-syn immunohistochemically reacted coronal sections of the human amygdala BL complex from samples from patients with Parkinson’s disease (**A**) and Huntington’s disease (**B**); scale bar = 2000 μm. Coronal sections of human BM (**C, F and I**), BL (**D, G and J**) and La (**E, H and K**) amygdaloid nuclei immunohistochemically stained for α-syn protein from samples from patients with Parkinson’s disease (control positive) (**C–E**), without disease (negative control) (**F–H**) and with Huntington’s disease (**I–K**). Scale bar (**C–K**) = 100 µm.

Since the amygdaloid complex has been described as a site for multiple pathologies, immunohistochemistry against different proteinopathies (Supplementary Table 1) confirmed the copathologies of neuropathological diagnosis with the presence of tau (Fig. 4), amyloid β (Fig. 5) and TDP43 (Fig. 6) but not α-synuclein (Fig. 7). Mosaics of the BL complex of patients positive for each proteinopathy (Figs. 4A and 5A and 6A and 7A) as well as patients with Huntington’s disease (Figs. 4B and 5B and 6B and 7B) were included. Images of each basolateral complex nucleus from control-positive patients (Figs. 4C–E, 5C–E, 6C–E and 7C–E), control-negative patients (Figs. 4F–H, 5F–H, 6F–H and 7F–H) and patients with Huntington’s disease (Figs. 4I–K, 5I–K, 6I–K and 7I–K) illustrate copathology in Huntington’s disease. Confocal images of HTT deposits did not reveal colocalization with either tau, amyloid β, TDP43 or α-synuclein (Supplementary Fig. 1). A qualitative analysis was carried out for the presence or absence of proteinopathies and did not reveal a relationship between Vonsattel grade and the presence of copathologies (Supplementary Table 4).

## Discussion

The main findings of the present report are summarized below. This study describes the distribution of HTT in the human BL amygdaloid complex in samples from patients with Huntington’s disease. The results did not reveal significant differences among the amygdaloid BL nuclei or as a function of the sex of the patient. The estimated HTT deposits did not correlate with the number of CAG repeats but were significantly and positively correlated with the Vonsattel stage. Additional aggregates of other pathological proteinopathies were also observed.

The main limitations of the present study include the difficulty of obtaining an elevated number of samples. The samples received were not from the same rostrocaudal or dorsoventral levels and thus did not always include all the nuclei of the BL complex. Genetic diagnosis, when available, was carried out from blood samples or from cerebral tissue with the corresponding discrepancies.

The classic neuropathological diagnosis of Huntington’s disease follows Vonsattel staging, which is based on macroscopic changes in the striatum and neurodegeneration and gliosis and is mostly based on classical techniques such as Nissl staining.^15^ Later, HTT deposits were identified using specific antibodies in areas beyond the striatum, such as the cerebral cortex.^21^ To our knowledge, the present report describes, for the first time, the distribution and quantification of HTT in the human BL amygdaloid complex. The results did not reveal significant differences among the BM, BL or La amygdaloid nuclei.

Although a significant disparity in motor symptoms has been reported between sexes, with females consistently presenting more symptoms than males as well as in terms of behavioural symptoms, specifically depressive symptoms,^37^ our results have not shown any differences among HTT in the amygdala that could reflect this different symptomatology.

In contrast with our results, inclusions in the cortex correlate with HTT repeat expansion but not with pathological grade.^21^ Interestingly, CAG repeats have been demonstrated to be relevant in tauopathies.^38^ Our results demonstrate that estimated HTT deposits are significantly and positively correlated with the Vonsattel stage (Fig. 2I). Notably, the clinical severity of Huntington’s disease does not always correlate with neuropathological stage.^39^ In fact, the age of onset of motor symptoms is inversely correlated with the length of the CAG triplet repeat,^40^ and the triplet repeat is the only main factor, as is the alteration of given genetic loci, such as FAN1, MSH3 or MLH1.^41^ Therefore, further research is needed to characterize the relationships among clinical severity, pathological grade, HTT deposits and CAG repeats.

The original pathological stage was established based on macroscopic and microscopic observations of gliosis and neurodegeneration via classical techniques such as Nissl staining in the caudate and putamen.^15^ Subsequently, more specific HTT deposit markers, including anti-p62^20^ and anti-HTT-specific^21^ antibodies showing intracellular and intraneuronal deposits, have been used. The antibody EM48, used in the cerebral cortex in the present study, detected deposits that were small, nonnuclear and more abundant in patients with Huntington’s disease with increased CAG repeat length, higher Vonsattel grade and earlier age of onset.^42^ Additionally, colocalization studies did not reveal HTT deposits in microglial or astroglial populations (Fig. 3). Recent approaches have revealed characteristic astroglial patterns in white matter.^43^

Although original descriptions focused on the striatum, pathology beyond the striatum has been identified; thus, this disease is now considered multisystemic.^20^ Many other regions of the limbic system,^25,44^ namely, the olfactory bulb^45^ and the amygdala,^28^ which are related to nonmotor symptoms such as hyposmia^46,47^ and depression,^48^ have only occasionally been investigated from a neuropathological perspective. Depression constitutes one of the earliest, and most devastating, symptoms associated with Huntington’s disease and has been reported to affect approximately half of all gene-positive Huntington’s disease family members.^49^ Disruption of the connectivity of the amygdala with other regions constitutes basic neural damage underlying this symptom.^26^

In fact, amygdala volume is extensively used as a diagnostic tool.^26,27,50,51^ Although stereological approaches are used to quantify pathology in different regions,^52-54^ to our knowledge, this report constitutes the first stereology-based quantification of HTT in the human amygdala. Furthermore, these results demonstrated a positive correlation between HTT deposits in the human amygdala and the Vonsattel stage.^15^

Interestingly, the amygdala has been defined as a ‘hub’ from a connectomic perspective^55^ or an ‘incubator’ for misfolded proteins in many neurodegenerative diseases,^31^ such as Alzheimer’s disease^56^ and Parkinson’s disease.^36,57^ Our results demonstrate that in Huntington’s disease patients, the copathology of other neurodegenerative diseases, such as Alzheimer’s disease, also occurs (Figs. 4–6). New approaches, including the prion-like view of HTT^14,58^ and the connectome perspective of amygdalo-striatal dysconnectivity,^59^ may provide new insights into the complex pathoetiology of Huntington’s disease, opening a new perspective in neuropathological diagnosis and improving the understanding of nonmotor symptoms such as depression.

## Supplementary Material

fcaf502_Supplementary_Data

## Data Availability

The data that support the findings of this study are available from the corresponding author upon reasonable request.
