# Supplementary material for: Huntingtin in the amygdaloid basolateral complex is correlated with Vonsattel staging in Huntington’s disease
Source: Brain Commun. 2025 Dec 22;8(1):fcaf502. doi: 10.1093/braincomms/fcaf502 (PMC12776364; doi:10.1093/braincomms/fcaf502)
Supplement: fcaf502_Supplementary_Data [file fcaf502_supplementary_data.pdf]

Supplementary Table 1. Estimated HTT population in the basomedial (BM), basolateral (BL) and lateral (La) nuclei.

	Case	Number of Sections	Section Cut Thickness (μm)	Section Evaluation Interval	Disector Height (Z) (μm)	Guard Zone Distance (μm)	Counting Frame Area (XY) (μm²)	Sampling Grid Area (XY) (μm²)	Number of Sampling Sites	Total Markers Counted	Estimated Population using Mean Section Thickness	Coefficient of Error (Gundersen) error m=1
BM	2	4	50	13	5	2	5,625	40,000	1,720	207	37,328	0.08
	7	5	50	13	5	2	5,625	40,000	1,979	317	54,951	0.06
	8	3	50	13	5	2	5,625	40,000	1,432	107	18,257	0.12
BL	1	3	50	13	5	2	5,625	40,000	2,103	279	49,128	0.06
	2	5	50	13	5	2	5,625	40,000	1,898	407	71,968	0.05
	3	5	50	13	5	2	5,625	40,000	8,761	928	163,995	0.04
	4	4	50	13	5	2	5,625	40,000	1,171	384	64,510	0.10
	5*	5	50	13	5	2	5,625	40,000	8,877	6,258	1,133,064	0.03
	6	5	50	13	5	2	5,625	40,000	3,048	583	98,252	0.05
	7	5	50	13	5	2	5,625	40,000	4,415	1,044	176.965	0.04
	8	4	50	13	5	2	5,625	40,000	1,611	130	22,975	0.09
	9	3	50	13	5	2	5,625	40,000	2,015	465	81,690	0.06
La	2	5	50	13	5	2	5,625	40,000	3,443	880	153,099	0.05
	3	4	50	13	5	2	5,625	40,000	3,124	264	47,963	0.07
	4	3	50	13	5	2	5,625	40,000	1,616	382	66,294	0.09
	6	2	50	13	5	2	5,625	40,000	371	48	8,021	0.15
	7	4	50	13	5	2	5,625	40,000	1,651	837	148,733	0.05
	8	4	50	13	5	2	5,625	40,000	2,044	153	26,215	0.09
	9	2	50	13	5	2	5,625	40,000	723	131	21,570	0.11

*Outliers

Supplementary Table 2. Estimated HTT population in the basolateral complex.

	Case	Vonsattel grade	Number of Sections	Section Cut Thickness (μm)	Section Evaluation Interval	Disector Height (Z) (μm)	Guard Zone Distance (μm)	Counting Frame Area (XY) (μm²)	Sampling Grid Area (XY) (μm²)	Number of Sampling Sites	Total Markers Counted	Estimated Population using Mean Section Thickness	Coefficient of Error (Gundersen) error m=1
**Male**	1	2	3	50	13	5	2	5,625	40,000	2,103	279	49,128	0.06
	2	3	5	50	13	5	2	5,625	40,000	7,061	1,494	262,588	0.04
	3	3	5	50	13	5	2	5,625	40,000	11,885	1,192	211,961	0.04
	5*	2	5	50	13	5	2	5,625	40,000	8,877	6,258	1,133,064	0.03
	7	4	5	50	13	5	2	5,625	40,000	8,045	2,198	380,440	0.03
	9	2	4	50	13	5	2	5,625	40,000	2,738	596	103,218	0.05
	11	4	4	50	13	5	2	5,625	40,000	3,840	2,835	492,442	0.04
**Female**	4	2	4	50	13	5	2	5,625	40,000	2,787	766	130,934	0.08
	6	2	5	50	13	5	2	5,625	40,000	3,419	631	106,269	0.05
	8	3	4	50	13	5	2	5,625	40,000	5,087	390	67,469	0.06

*Outliers

Supplementary Table 3. Details of antibodies used in this study.

	Antigen	Manufacturer	Catalog nº	Host	Bloking buffer	Incubation	Secondary antibody
Immunohistochemistry	Huntingtin (clone mEM48)	Sigma–Aldrich (St Louis, USA)	MAB5374	Mouse monoclonal	PBS+0.4% TX-100+10% NHS	1:50 in PBS+0.4% TX-100+10% NHS (4°C, overnight)	1:200 Biotinylated horse anti-mouse Ig G (H+L) Vector laboratories
	Tau (Tau46) mAb	Cell Signaling (Massachusetts, USA)	#4019	Mouse monoclonal	PBS+0.3% TX-100+5% NHS	1:1500 in PBS+10% TX-100 (4°C, overnight)	
	Amyloid Beta x-42 (clone 12F4)	Sigma–Aldrich (St Louis, USA)	05-831-I	Mouse monoclonal	PBS+0.3% TX-100+5% NHS	1:5000 in PBS+0.3% TX-100 (4°C, overnight)	
	α-Synuclein	Novocastra (Newcastle, UK)	NCL-L-ASYN	Mouse monoclonal	-	1:20 in PBS in PBS+0.3% TX-100 (4°C, 72 h)	
	Phospho-TDP-43 (Ser409, Ser410)	Invitrogen (Massachusetts, USA)	PA5-114661	Rabbit polyclonal	PBS+0.3% TX-100+10% NHS	1:100 in PBS+0.3% TX-100+5% NHS (4°C, overnight)	1:200 Biotinylated horse anti-rabbit Ig G (H+L) Vector laboratories
Immunofluorescences	Huntingtin (clone mEM48)	Sigma–Aldrich (St Louis, USA)	MAB5374	Mouse monoclonal	PBS+0.4% TX-100+10% NHS+0.4% BSA	1:50 in PBS+0.3% TX-100 (4°C, overnight)	1:200 Alexa Fluor® 568 donkey anti-mouse IgG (H+L) Invitrogen
	GFAP	Abcam (Cambridge, USA)	ab53554	Goat polyclonal	PBS+0.4% TX-100+10% NHS+0.4% BSA	1:500 in PBS+0.3% TX-100 (4°C, overnight)	1:200 Alexa Fluor® 647 donkey anti-goat IgG (H+L) Invitrogen
	Iba-1	Abcam (Cambridge, USA)	ab5076	Rabbit polyclonal	PBS+0.4% TX-100+10% NHS+0.4% BSA	1:1000 in PBS+0.3% TX-100 (4°C, overnight)	1:200 Alexa Fluor® 488 donkey anti-rabbit IgG (H+L) Invitrogen
	Huntingtin (clone mEM48)	Sigma–Aldrich (St Louis, USA)	MAB5374	Mouse monoclonal	-	1:50 in PBS+0.4% TX-100+10 % NHS (4°C, overnight)	1:200 Alexa Fluor® 568 donkey anti-mouse IgG (H+L) Invitrogen
	Tau (D1M9X) XP® mAb	Cell Signaling (Massachusetts, USA)	46687S	Rabbit polyclonal	-	1:1500 in PBS+0.4% TX-100+10 % NHS (4°C, overnight)	1:200 Alexa Fluor® 488 donkey anti-rabbit IgG (H+L) Invitrogen
	Beta-Amyloid	Cell Signaling (Massachusetts, USA)	2454S	Rabbit polyclonal	-	1:5000 in PBS+0.4% TX-100+10 % NHS (4°C, overnight)	
	Phospho-TDP-43 (Ser409, Ser410)	Invitrogen (Massachusetts, USA)	PA5-114661	Rabbit polyclonal	-	1:100 in PBS+0.4% TX-100+10 % NHS (4°C, overnight)	
	Huntingtin (clone mEM48)	Sigma–Aldrich (St Louis, USA)	MAB5374	Mouse monoclonal	-	1:50 in PBS+0.3% TX-100 (4°C, overnight)	1:200 Alexa Fluor® 568 donkey anti-mouse IgG (H+L) Invitrogen
	α-synuclein (phospho S129)	Abcam (Cambridge, USA)	ab59264	Rabbit polyclonal	-	1:100 in PBS+0.3% TX-100 (4°C, overnight)	1:200 Alexa Fluor® 488 donkey anti-rabbit IgG (H+L) Invitrogen

Supplementary Table 4. Analysis of the presence (+) or absence (-) of other pathological proteins in the human amygdaloid basolateral complex in Huntington's disease.

Case	Neuropathological diagnosis	Vonsattel grade	Age (years)	Tau	Amyloid β	TDP43	α-synuclein
1	HD	2	72	+ *	+	+	n.a.
2	HD	3	72	+	n.a.	+	n.a.
3	HD	3	70	n.a.	+ *	+	n.a.
4	HD	2	72	-	+	n.a.	n.a.
5	HD	2	70	+	+	+	n.a.
6	HD	2	72	n.a.	-	+	n.a.
7	HD	4	64	+	-	+	n.a.
8	HD	3	68	n.a.	n.a.	+ *	+ *
9	HD	2	53	n.a.	n.a.	+	n.a.
16	C		43	-	n.a.	n.a.	-
17	C		58	n.a.	-	+ *	n.a.
18	C		58	+ *	-	+	- *
19	C		65	+	n.a.	n.a.	-
20	C		81	n.a.	- *	+	n.a.
21	PD		82	n.a.	n.a.	n.a.	+ *
22	AD		71	+ *	+ *	n.a.	n.a.
23	AD		89	n.a.	n.a.	+ *	n.a.

AD: Alzheimer's disease, HD: Huntington's disease, n.a.: not analysed, PD: Parkinson's disease, +: presence, -: absence; *: case shown in the corresponding figure.

Supplementary Figure 1

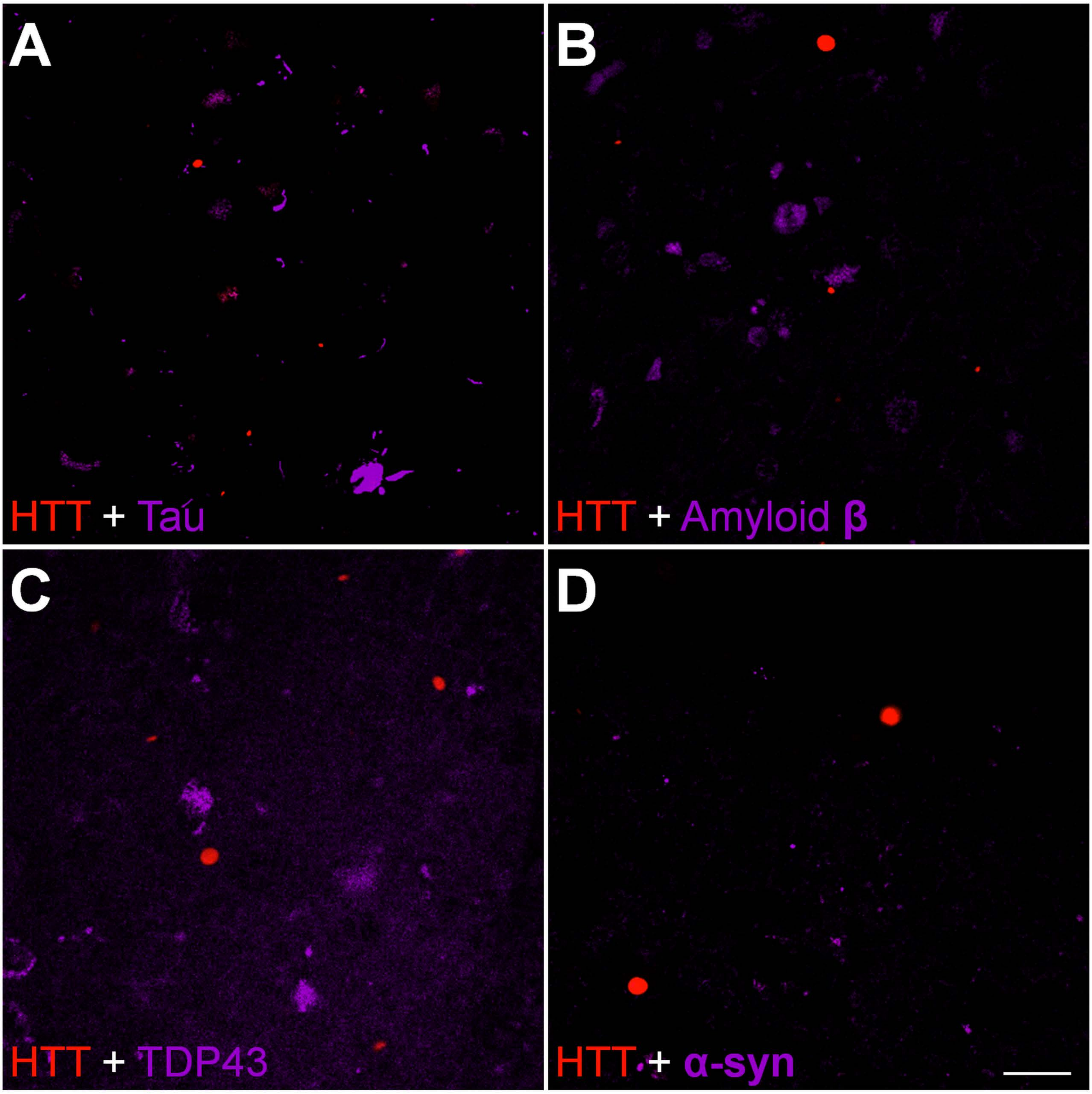

Relationship of HTT with other pathological proteins. Confocal microscopy images showing double immunofluorescence in the human basolateral amygdaloid complex in Huntington's disease. The relationships between HTT deposits (A-D) and other pathological proteins such as Tau (A), Amyloid β (B), TDP 43 (C) and α-syn (D) are shown. Scale bars A: 40 μm; B-D: 20 μm.
